# Comparative Evaluation of Waist-to-Height Ratio and BMI in Predicting Adverse Cardiovascular Outcome in People With Diabetes: A Systematic Review

**DOI:** 10.7759/cureus.38801

**Published:** 2023-05-09

**Authors:** Ajoy Tewari, Gaurav Kumar, Anuj Maheshwari, Vineeta Tewari, Jay Tewari

**Affiliations:** 1 Diabetes and Endocrinology, Jai Clinic and Diabetes Care Center, Lucknow, IND; 2 Department of Conservative, Endodontics and Aesthetic Dentistry, Dental Institute, Rajendra Institute of Medical Sciences (RIMS), Ranchi, IND; 3 Department of Medicine, Hind Institute of Medical Sciences, Lucknow, IND; 4 Department of Medicine, Shri Hari Kamal Diabetes and Heart Clinic, Lucknow, IND; 5 Department of Anatomy, Era's Lucknow Medical College and Hospital, Era University, Lucknow, IND; 6 Department of Internal Medicine, King George's Medical University (KGMU), Lucknow, IND

**Keywords:** adverse cardiovascular outcomes, cardiovascular risk, waist height ratio, body mass index, central obesity, diabetes

## Abstract

Central obesity is strongly associated with cardiovascular risk in people with diabetes. BMI does not reflect a regional fat distribution. The other anthropometric indices, which are markers of central obesity, like waist circumference and waist-hip ratio, are subject to age, sex, and ethnic variations. An index like waist-to-height ratio (WHtR), which considers central obesity, outperforms BMI in predicting cardiometabolic risk. With a single cut-off of 0.5, irrespective of age, sex, and ethnic variations, WHtR has a wide application in screening obesity in population settings. Previous systematic analyses were conducted in the general population, assessing cardiometabolic risk. The current study is the first systematic analysis to compare the applicability of WHtR and BMI in predicting both cardiovascular risk and adverse cardiovascular outcomes in people with diabetes. It includes prospective cohort studies, cross-sectional studies, and randomized control trials to generate evidence. The summary scores indicate that WHtR is probably a better indicator than BMI to assess cardiovascular risk in people with diabetes. A future meta-analysis will pave the way for more robust evidence.

## Introduction and background

Central adiposity contributes to adverse outcomes in people with type 2 diabetes mellitus. Adverse cardiovascular (CV) outcomes contribute maximally to mortality in people with type 2 diabetes mellitus. Waist-to-height ratio (WHtR) is emerging as a better indicator than BMI to assess obesity, especially in Southeast Asians, because of the high prevalence of central adiposity and predisposition to coronary artery disease [1]. An anthropometric index, to be used as a public health screening tool, should have well-defined cut-offs, which can be used in different ethnic, age, and sex groups. BMI measurement cannot encompass the entire spectrum of adiposity, like normal-weight central obesity and metabolically healthy obesity. Further, it may not show the relative adipose tissue and lean body mass content. It is not an index in a genuine sense because an index ought to be dimensionless, and its further limitation is that increments in mass are instead in a cube than the square of the height [2]. Assessment of the waist-to-hip ratio in practical risk management is not advantageous, as weight loss results in the reduction of waist and hip size, without altering its ratio. Measuring waist circumference (WC) without height adjustment may lead to overestimating fat mass in tall subjects and underestimating in short individuals [3]. WC requires population-specific values for various ethnicities [4]. WHtR is the ratio of waist circumference and the individual's height. It is easy to report, not being affected by usual factors such as age, sex, and ethnicity, and it negates the use of scales [5]. It is simple to report that a value of <0.5 or >0.5 defines obesity. Therefore, the WHtR, with a recommended value of 0.5 (maintaining one's waist circumference at less than half of one's height), serves as a simple public health message [6]. The concept that WHtR can predict coronary heart disease came from Japanese researchers in 1995 [7, 8]. WHtR, like WC, is highly correlated with abdominal fat measured by ‌imaging techniques [9]. WHtR for central obesity was found to be a better predictive marker of "early health risk" than BMI in the general population [10, 11]. There is no comprehensive investigation that links WHtR and BMI to worse CV outcomes in diabetics. In this research, the evidence for using WHtR, a measure of central adiposity, as a predictor of cardiovascular disease (CVD) and related risk factors in individuals with diabetes is carefully reviewed. The review uses data from prospective cohorts, cross-sectional studies, and randomized control trials that indicate links between worse CV outcomes, WHtR, and BMI in people with diabetes to frame the linkages.

## Review

Electronic databases, including PubMed (Table 1), Embase, and Cochrane Central, were systematically searched for available literature. Combining categories (to select papers, including WHtR and BMI) yielded 340 articles in PubMed. A search in the Cochrane and Embase databases yielded 173 and 64 papers, respectively. A total of 577 papers were identified from three databases, and 20 duplicates were removed. Two reviewers (Tewari A and Kumar G) independently evaluated the eligibility of these 557 articles using the predetermined inclusion and exclusion criteria. Inclusion criteria were studies using WHtR and BMI in people with type 2 diabetes mellitus to predict adverse cardiovascular outcomes. There was no limitation on the country of origin or study setting, including both in-hospital and non-hospital environments. Studies in children, gestational diabetes mellitus (GDM), and type 1 DM were excluded.

**Table 1:** Search strategy for the literature search in the electronic database.

Database	Search Strategy
PubMed	("body mass index"[MeSH Terms] OR ("body mass index"[MeSH Terms] OR ("body"[All Fields] AND "mass"[All Fields] AND "index"[All Fields]) OR "body mass index"[All Fields]) OR "bmi"[All Fields] OR "body weight"[MeSH Terms] OR "body weights and measures"[MeSH Terms] OR ("weight s"[All Fields] OR "weighted"[All Fields] OR "weighting"[All Fields] OR "weightings"[All Fields] OR "weights and measures"[MeSH Terms] OR ("weights"[All Fields] AND "measures"[All Fields]) OR "weights and measures"[All Fields] OR "weight"[All Fields] OR "body weight"[MeSH Terms] OR ("body"[All Fields] AND "weight"[All Fields]) OR "body weight"[All Fields] OR "weights"[All Fields]) OR "body height"[MeSH Terms] OR ("body height"[MeSH Terms] OR ("body"[All Fields] AND "height"[All Fields]) OR "body height"[All Fields] OR "height"[All Fields] OR "heights"[All Fields]) OR ("ratio"[All Fields] OR "ratio s"[All Fields] OR "ratioes"[All Fields] OR "ratios"[All Fields])) AND ("waist height ratio whtr"[All Fields] OR "waist height ratio"[All Fields] OR "waist stature ratio"[All Fields] OR "waist height ratio"[MeSH Terms]) AND (("type2"[All Fields] AND ("diabete"[All Fields] OR "diabetes mellitus"[MeSH Terms] OR ("diabetes"[All Fields] AND "mellitus"[All Fields]) OR "diabetes mellitus"[All Fields] OR "diabetes"[All Fields] OR "diabetes insipidus"[MeSH Terms] OR ("diabetes"[All Fields] AND "insipidus"[All Fields]) OR "diabetes insipidus"[All Fields] OR "diabetic"[All Fields] OR "diabetics"[All Fields] OR "diabets"[All Fields])) OR "diabetes mellitus, type 2"[MeSH Terms]) AND ("cardiovascular risk"[All Fields] OR "cardiovascular diseases/complications"[MeSH Terms] OR "cardiovascular disease"[All Fields] OR "cardiovascular diseases"[MeSH Terms] OR "myocardial infarction"[All Fields] OR "myocardial infarction"[MeSH Terms] OR "coronary disease"[All Fields] OR "coronary disease"[MeSH Terms] OR "death, sudden, cardiac"[MeSH Terms] OR "coronary artery disease"[MeSH Terms] OR "cardiovascular risk"[All Fields] OR "cardiovascular risk"[Title/Abstract])

**Figure 1:**
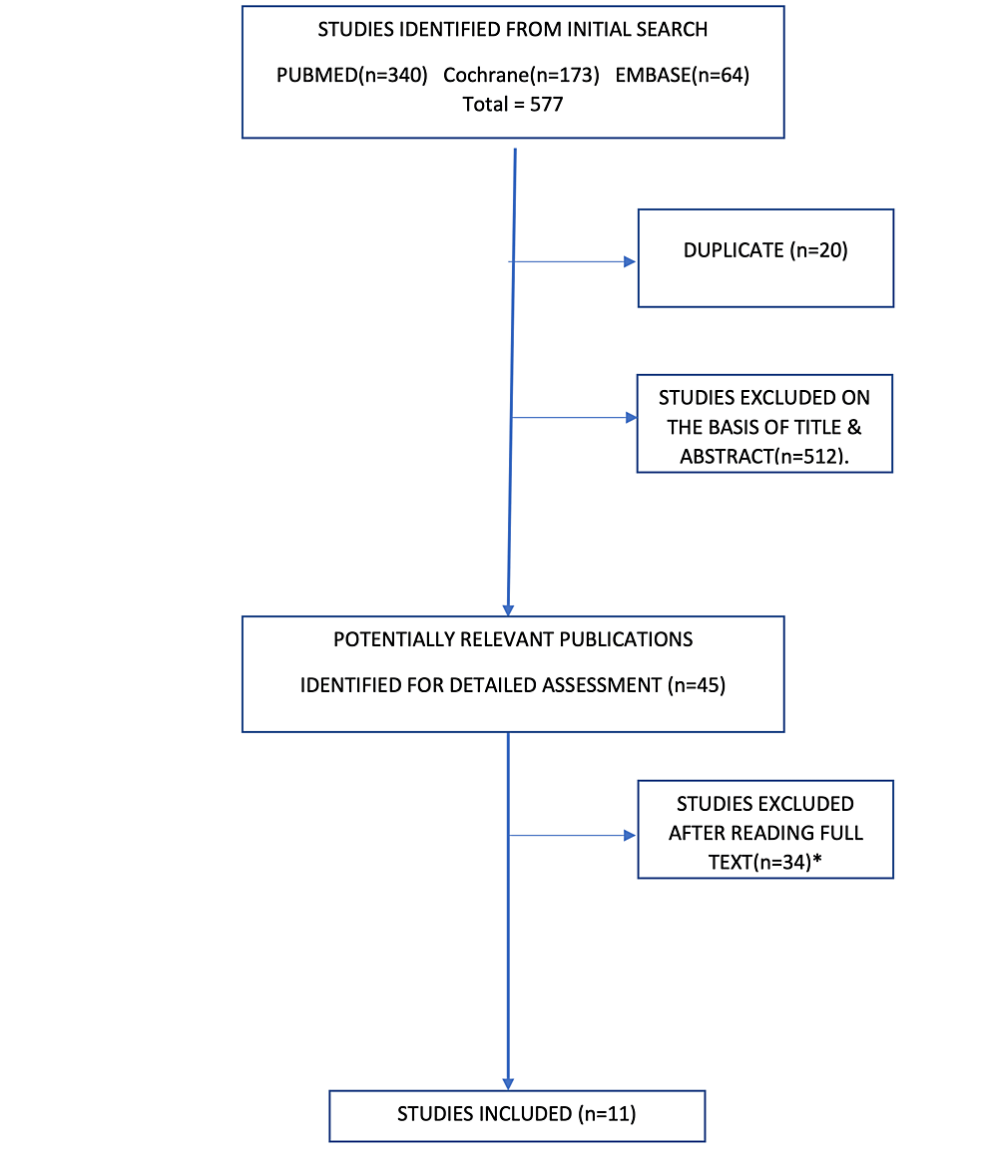
PRISMA flowchart. *Reasons for exclusion: 20 studies were in the general population, not in people with diabetes; in eight studies, subgroup analysis of people with diabetes was not available; three studies had an improper selection of intervention and control group as per protocol; and three studies assessed non-CV outcomes (outcomes not as per the protocol). PRISMA: Preferred Reporting Items for Systematic Reviews and Meta-Analyses.

Selected articles for inclusion in the current review are displayed in the search results (Figure 1). A total of 557 articles were identified after an electronic search, out of which 512 articles were excluded following title and abstract screening. Forty-five potentially relevant articles were identified for full-text review. A total of 34 papers were found unsuitable after full-text review for the following reasons: 20 studies were in the general population, not in people with diabetes (population not as per protocol); in eight studies, subgroup analysis of people with diabetes was not available; three studies had an improper selection of intervention and control group as per protocol; and three studies assessed non-CV outcomes (outcomes not as per the protocol). Finally, 11 papers met the inclusion criteria and were included in the systematic review [12-22].

The quality of the included studies was assessed using the Newcastle-Ottawa Scale (NOS) for non-randomized and cross-sectional studies in meta-analyses. In contrast, the risk of bias (RoB) for randomized controlled trials was assessed using the Cochrane RoB tool. The NOS evaluates the quality of non-randomized studies based on three broad categories: selection of study groups, comparability of study groups, and assessment of outcome or exposure. The tool assigns a certain number of stars to each study based on its quality, with a maximum of nine stars possible.

Results

Performance of Adiposity Factors to Predict Cardiovascular Outcomes

Many studies assessing cardiometabolic risk compared WHtR and BMI in the general population [5]. However, most of them lack subgroup analyses for people with diabetes. In the current review, eight studies were primarily done on people with diabetes, and in three studies, subgroup analyses of people with diabetes were performed. The present systematic analysis analyzed data from four prospective cohort studies with total subjects of 16672, six cross-sectional observational studies with 5394 subjects, and one randomized control trial with 11125 subjects. Eight studies were performed primarily in people with diabetes, while three studies were subgroup analyses in people with diabetes. Studies included other markers of generalized obesity and central obesity like waist circumference, WHtR, hip circumference, waist circumference, fat mass (FM), body fat percentage (BF%), free fat mass (FFM), total body water (TBW), wrist circumference, body adiposity index, abdominal volume index, visceral adiposity index, body roundness index, Conicity index, and body adiposity index. However, only those studies in which both WHtR and BMI were measured were included in the review. Cross-sectional outcome measures considered in the present review are any of the risk factors related to CVD, sudden cardiac death, NT-proBNP, asymmetric dimethylarginine (ADMA), endothelin 1 (ET-1), CV risk by Framingham Risk Scores (FRS), the UKPDS2.0, the ADVANCE risk engines, stroke, cardio-ankle vascular index (CAVI), brachial-ankle pulse wave velocity (baPWV), hypertension atherogenic dyslipidemia, metabolic syndrome (MetS), high total cholesterol (TC), high triglyceride (TG), low high-density lipoprotein cholesterol, the composite of death from CVD, non-fatal myocardial infarction, non-fatal stroke, and secondary outcomes such as myocardial infarction, stroke, CV death, and death from any cause.

Interpretation of Included Studies

Moazzeni SS et al. [12] showed no significant association of BMI with sudden cardiac death in people with and without type 2 DM but a significant association with the WHtR in people with diabetes and without type 2 DM. ADMA, endothelin 1 (ET-1), and NT-proBNP, as well as body composition parameters (BF%, FM, FFM, and TBW), were investigated in a study by Markova A et al. [13] to see if they were associated with calculated CV risks. It was discovered that the strongest correlations between the calculated CV risk estimations were found with BMI, WHtR, and BF%. FM, FFM, and TBW performed much worse simultaneously and were exclusively linked to stroke risks. While ADMA exhibited correlations to WHtR, FFM, and TBW, and ET-1 correlated with BMI and FM alone, levels of NT-proBNP were unrelated to any measure of obesity. 

Gomez-Sanchez L et al. [14] used CAVI and baPWV to assess CV risk. It had a statistically significant (p<0.01) negative association with BMI, WHtR, and other adiposity markers. CAVI variability with BMI, WHtR, and other adiposity indicators was more pronounced in people with diabetes. Guasch-Ferré M et al. [15] did an interesting cross-sectional study seeking a correlation between adiposity markers and hypertension, atherogenic dyslipidemia, and MetS. The area under the curve (AUCs) for WHtR and WC were significantly higher than AUCs of BMI for atherogenic dyslipidemia and MetS; conversely, BMI was the strongest predictor of hypertension. In this study, WHtR and WV showed significance for atherogenic dyslipidemia compared to BW. WHtR and WC had a positive association with metabolic syndrome compared to BMI, with significantly higher AUCs and a more than 70% predictive capability.

According to the findings from the ADVANCE ON trial [16], a randomized control trial focusing primarily on people with type 2 diabetes, the hazard ratio for a significant macrovascular event was 1.16 for WHtR, which was one SD higher, and 1.09 for BMI. There was no gender or geographic heterogeneity, but older adults (aged 66 or older) were more significantly affected by this risk. There was also proof that WHtR performed just marginally better in major CV event prediction than BMI and WHtR. There was no evidence of a J-shaped curve in the relationship between WHtR and the risk of a major macrovascular incident for a BMI range of 18.5-40 kg/m2 and a WHtR range of 0.48-0.74. Regarding location, sex, and age, WHtR did not change. For the increased risk of CVD in this group of type 2 diabetics, a value of 0.55 could be more acceptable. With a WHtR of 0.55 compared to a WHtR of 0.55, the study found an elevated risk for a major cardiovascular event, CV mortality, and death from any cause, but not for myocardial infarction and stroke. With the exception of stroke in those older than 66 years, all subgroups showed an elevated risk of CVD when the WHtR was 0.6. WHtR cut-offs with higher values are, therefore, more prognostic of CVD in diabetics. In the study by Obirikorang C et al. [17], the researchers investigated the relationship between adiposity indicators and hypertension, high TC, high TG, low HDL-C, and METS as defined by the International Diabetes Federation (IDF). In addition, they observed that WHtR and BMI were significantly associated with hypertension and METS-IDF. In a prospective cohort study, Lim RB et al. [18], in a prospective cohort study, correlated adiposity markers with all-cause and CV mortality. BMI is not positively associated with short-term mortality. At the same level of BMI, both the fourth and highest quintiles of WHtR were significantly associated with increased risk of all-cause or CVD mortality.

In a cross-sectional study, Li P et al. found that as WHtR, WC, and BMI increased, the likelihood of achieving the combined therapeutic goals for BP, glucose, and lipid levels decreased. For people with a WHtR greater than 0.59, the likelihood of attaining all three targets was lower (p<0.005) after adjusting for confounders [19]. Tonding SF et al. [20], in a cross-sectional study, found no significant association between WHtR and CV risk scores. However, they found a significant association between the conicity index and body adiposity index. In prospective cohort research, Khalili S et al. [21] discovered that WHtR was moderately linked with incident CVD in both genders. An increase in WHtR of 1 SD increased CVD risk in men and women by 19% and 18%, respectively.

Using the targeted maximum likelihood estimation (TMLE) method, Mozafar Saadati H et al. [22] designed a prospective cohort study to investigate the unbiased association of BMI and central obesity with the risk of stroke separately for diabetics and nondiabetics in the Atherosclerosis Risk in Communities (ARIC) cohort study. The results show that the effect of BMI in people with diabetes was more attenuated in the full model (RR: 1.04 [0.90, 1.20]) than in nondiabetics (RR: 1.11 [1.00, 1.24]) for all participants after adjusting for demographic, behavioral, and biological covariates and central obesity indices. The effects for other indices, aside from WHtR, were marginally significant. WHtR provided some protection, although it was little. The studies included in the current review have used various anthropometric indices to assess CV risk in people with diabetes. WHtR appears to have better predictability than BMI for cardiovascular risk in people with diabetes. The quality of the included studies is depicted in Tables 2-4. 

**Table 2:** The Newcastle-Ottawa Scale (NOS) for assessing the quality of non-randomized studies in meta-analysis.

Criteria	Moazzeni SS et al. [12]	Khalili S et al. [21]	Lim RB et al. [18]	Mozafar Saadati H et al. [22]
Selection				
Representativeness of the exposed cohort	*	*	*	*
Selection of the nonexposed cohort	*	*	*	*
Ascertainment of exposure	*	*	*	*
Demonstration that outcome of interest was not present at start of study	*	*	*	*
Comparability				
Comparability of cohorts on the basis of the design or analysis	**	**	**	**
Outcome				
Assessment of outcome	*	*	*	*
Was follow-up long enough for outcomes to occur	*	*	*	*
Adequacy of follow-up of cohorts (<20%)	*	*	*	*
Total awarded stars	9 star	9 star	9 star	9 star

**Table 3:** The Newcastle-Ottawa Scale (NOS) for assessing the quality of cross-sectional studies.

Criteria	Markova A et al. [13]	Gomez-Sanchez L et al. [14]	Guasch-Ferré M et al. [15]	Obirikorang C et al. [17]	Li P et al. [19]	Tonding SF et al. [20]
Selection						
Truly representative of average in target population	-	*	*	-	*	-
Sample size	*	*	*	*	*	-
Non-respondents	*	*	*	*	*	*
Ascertainment of the exposure (WHtR)	**	**	**	**	**	**
Comparability						
Comparability of different outcome group on the basis of the design or analysis	**	**	**	**	**	**
Outcome						
Assessment of outcome	**	**	**	**	**	**
Statistical tests	*	*	*	*	*	*
Total awarded stars	9 star	10 star	10 star	9 star	10 star	8 star

**Table 4:** Summary of the risk of bias assessment of the included randomised controlled trial.

Criteria	Rådholm K et al. [16]
Random sequence generation (selection bias)	Unclear risk
Allocation concealment (selection bias)	Unclear risk
Blinding of participants and personnel (performance bias)	High risk
Blinding of outcome assessment (detection bias)	Unclear risk
Incomplete outcome data (attrition bias)	Low risk
Selective reporting (reporting bias)	Low risk
Other bias	Low risk

Discussion

The characteristics of the included studies are summarized in Table 5. In the absence of a complete meta-analysis, the "summary scores" in Table 6 represent our attempt to summarise the outcomes of the different studies included in the systematic analysis. The summary scores point towards the superiority of WHtR over BMI in predicting adverse outcomes in people with diabetes. A systematic review's findings also depend on the caliber of the included studies. The advantage of any systematic review is that it is a thorough, open, and inclusive procedure that eliminates a lot of other potential sources of bias that can occasionally be detected in narrative reviews.

**Table 5:** Characteristics of the included studies. WHtR: Waist-to-height ratio; NT-proBNP: N-terminal brain natriuretic pro-peptide; ADMA: Asymmetric dimethylarginine; ET1: Endothelin 1; FFM: Fat-free mass; BF%: Body fat percentage; FM: Fat mass; SCD: Sudden cardiac death; CAVI: Cardio-ankle vascular index; CVD: Cardiovascular disease.

Study reference	Study design	Follow-up duration (years)	Main cardiac outcome measure	Number of persons with outcome	Analysis type	Subjects (n)	Country	Age (year)	Sex	Result	Other obesity markers assessed	Study performed primarily in people with diabetes (P) or subgroup analysis (S)
Moazzeni SS et al. [12]	Prospective cohort study	15.8 (10.3-18.3)	Sudden cardiac death (SCD)	86	HR	1185	Iran	55.5	503M	No significant association of BMI with SCD in both people with and without type 2 DM, but significant association with waist-hip ratio in people with diabetes and WHtR in people without type 2 DM.	Waist circumference (WC), waist-to-hip ratio (WHR), and hip circumference (HC)	P
Markova A et al. [13]	Cross-sectional observational study	NA	NT-proBNP, ADMA, ET-1, CV risk by Framingham risk scores (FRS), the UKPDS2.0, and the ADVANCE risk engines	NA	Regression analysis	169	Bulgaria	60.3	102 F,67M	While FM, FFM, and TBW fared much worse and were solely related to the risks for stroke, BMI, followed by the WHtR and the BF%, exhibited the highest relationships with the estimated cardiovascular risk estimates. In contrast to ADMA, which showed linkages to WHtR, FFM, and TBW, and ET-1, which showed links to BMI and FM alone, levels of NT-proBNP were not connected to any measure of obesity.	Fat mass (FM), body fat percentage (BF%), free fat mass (FFM), total body water (TBW)	P
Gomez-Sanchez L et al. [14]	Cross-sectional observational study	NA	Cardio-ankle vascular index (CAVI), brachial-ankle pulse wave velocity (baPWV)	NA	Multiple linear regression analysis	2354,791 people with diabetes	Spain	61.4	463 M, 328F	CAVI and baPWV negative association with all adiposity measurements (p<0.01) for all; in the multiple linear regression, the proportion of CAVI variability by adiposity measures was higher among people with diabetes.	Body roundness index	S
Guasch-Ferré M et al. [15]	Cross-sectional observational study	NA	Hypertension, atherogenic dyslipidemia, MetS	NA	Multiple linear regression analysis	7,44,73,616	Spain	67	No data	AUCs for WHtR and WC were significantly higher than AUCs of BMI for atherogenic dyslipidemia and METS; conversely, BMI was the strongest predictor of hypertension.	Waist circumference	S
Rådholm K et al. [16]	Randomised control trial	9	Composite of death from CVD, nonfatal myocardial infarction, nonfatal stroke; secondary outcome: myocardial infarction, stroke, cardiovascular death, death from any cause	2162	Cox proportional hazard regression models	11125	20 countries	55 or higher	42% F,58% M	There was no gender or region-specific variability in the hazard ratio for a major macrovascular event, which was 1.16 for a one standard deviation higher WHtR and 1.09 for a higher BMI. However, persons over the age of 66 saw a larger impact. There is evidence that WHtR predicts major cardiovascular events just slightly better than BMI and WHR.	Waist circumference, waist-to-hip ratio	P
Obirikorang C et al. [17]	Cross-sectional observational study	NA	Hypertension, High TC, High TG, Low HDL-C, METS-IDF	NA	ROC and AUC	384	Ghana		147M, 237W	Both WHtR and BMI had significant associations with hypertension and METS-IDF.	Wrist circumference, body adiposity index, abdominal volume index, visceral adiposity index	P
Lim RB et al. [18]	Prospective cohort study	2.9	All-cause and CVD mortality	524	Cox proportional hazard regression models, competing risk models	13278	Singapore	Not specified	6680M,6208F	BMI is not positively associated with short-term mortality. At the same level of BMI, both fourth and highest quintiles of WHtR were significantly associated with increased risk of either all-cause or CVD mortality.	Waist circumference, waist-to-hip ratio	P
Li P et al. [19]	Cross-sectional study	NA	Guideline-recommended targets for HbA1c, BP, LDLc levels	NA	Logistic regression to determine odds ratio	3014	China	65.7	1526M, 1515F	In all eligible patients, with increasing WHtR, WC, and BMI, the combined therapeutic goal of attainment of BP, glucose, and lipid decreased. For people with WHtR greater than 0.59, the likelihood of attaining three targets was lower (p<0.005) after adjusting for confounders.	Waist circumference	P
Tonding SF et al. [20]	Cross-sectional study	NA	Cardiovascular risk assessment using UKPDS engine	NA	Multiple logistic regression	420	Brazil	61.9+- 9.5	53.5% F	No significant association of WHtR with cardiovascular risk scores.	Waist-to-hip ratio, Conicity index, body adiposity index	P
Khalili S et al. [21]	Prospective cohort study	8.4 years	First CVD events including definite MI, probable MI, unstable angina, angiographically proven coronary heartdisease and death from CVD	188	Cox proportional hazard model	1010	Iran	54.8 years	411M 599F	Independent of cofounders, WHtR was modestly linked with incident CVD in both sexes, and a one standard deviation rise in WHtR was associated with a 19% and 18% increase in CVD risk in men and women, respectively.	Waist-to-hip ratio, waist circumference	P
Mozafar Saadati H et al. [22]	Prospective cohort study	27	Definite or probable stroke	10,78,202	Targeted maximum likelihood estimation (TMLE)	1199	Iran	45-75	No data	The results (after controlling for demographic, behavioral, and biological covariates as well as central obesity indices as necessary) for all participants demonstrate that the effect of BMI in diabetics was more attenuated in the full model (RR: 1.04 [0.90, 1.20]) than in non-diabetics (RR: 1.11 [1.00, 1.24]), with the exception of WHtR. The protective effects of WHtR were minimal.	Body roundness index, body shape index, waist circumference, waist-to-hip ratio	S

**Table 6:** Performance of adiposity factors to predict cardiovascular outcomes. RCT: Randomized control trials; WHtR: Waist-to-height ratio.

Study Types	BMI	WHtR	Both	Partial WHtR
Cohort	¼	2/4		1/4
RCT		1/1		
Cross-sectional	1/6	1/6	4/6	

Prospective cohort studies included in the current systematic analysis show that WHtR outperformed BMI in predicting CVD. Results from cross-sectional studies were a mixed bag, with four studies showing a significant association between BMI and WHtR and one each in favor of BMI or WHtR. In our systematic analysis's only randomized control trial, WHtR outperformed BMI in predicting three-point major adverse cardiovascular events (MACE). From this, as per the level of evidence of the included studies, we suggest ‌WHtR would be a good screening tool. A systematic review and meta-analysis paved the way for using a cut-off of 0.5 to predict cardiometabolic risk in the general population [6]. Six reasons why the WHtR is a quick and reliable global indication of the health risks of obesity are listed in their study [5]. The reasons in favor of WHtR include better accuracy, the same boundary across all ethnicities and all age groups, and it can be depicted in a consumer-friendly chart. WHtR has been suggested as a good method for determining abdominal obesity since it may be used to adjust waist circumference for an individual's height. Moreover, CVD and height have been shown to be inversely correlated, and it is crucial to adjust for height in anthropometric measurements [23, 24]. The significance of this point is further underlined by the fact that adult height remains largely constant, so WHtR will change only when the waist measurement changes, as opposed to other indices like waist-hip ratio (WHR), which are more sensitive to changes in body size and could therefore increase or decrease both the hip and waist proportionately. Also, the evidence presented in the current systematic analytic summary shows that WHtR is likely a more accurate diagnostic predictor than BMI or WC. A meta-analysis is necessary to provide additional statistical evidence supporting this claim. This, however, is outside the purview of the current systematic study. The current systematic study discovered a trend in favor of 0.6, which was pragmatically suggested as a threshold value for increased risk [25], notably in people with diabetes. Another systematic review tried to answer the association of WHtR vis-à-vis BMI to predict coronary artery disease [10]. In another systematic review of the general population, WHtR outperformed BMI in predicting metabolic syndrome and diabetes, CV risk, and CV and all-cause mortality. The predictive power of WHtR was more in the Asian population [26]. A systematic review of the elderly population showed an association between WHtR and diabetes, metabolic syndrome, and CV risk compared to BMI and other parameters [27]. All these studies were conducted in the general population but gave insight into the use of WHtR in people with diabetes to predict CV risk. Four of the studies included in this systematic analysis were in Asian countries; they replicate the results in studies with Caucasian populations, signifying that the concept of WHtR holds equally good in all ethnicities. Further, it may be more useful in Asians and, more specifically, Southeast Asian ethnicities due to more visceral fat attributable to their unique genotype and phenotype.

Limitations 

The heterogeneity of the studies absolves the probability of conducting a meta-analysis. Further, it is challenging to compare anthropometric measurements to predict cardiovascular outcomes in a randomized control trial because of the confounding factors. We are aware that the "summary scores" used in the current systematic analysis in the absence of a meta-analysis have limits and that the statistical significance is influenced by a variety of variables, including the size of the population being studied and the inclusion of demographic or physiological adjustment variables. A systematic review's findings also depend on the caliber of the included studies. We admit that publication bias may have impacted the current findings. We have only included studies that were published in English. Some studies with different results may not have been submitted by authors, and other planned research with different results may not have been accepted by publishers.

## Conclusions

The current analysis is the first thorough examination of the data in favor of using WHtR, a stand-in for abdominal fat, as a predictor of CV risk in people with diabetes. It uses data from six cross-sectional studies, one randomized control trial, and four prospective cohort studies. Additionally, it contextualizes the link between WHtR and CV risk in reference to other obesity proxies like BMI and other measurements like hip circumference and WHtR. Data from many extremely large, nationally representative cohorts from different ethnic groups were included in the systematic study. WHtR, WC, and BMI are predictors of CVD and associated conditions, according to observations from studies included in our systematic review. WHtR is a more accurate predictor of adverse CV outcomes than BMI in individuals with diabetes. However, the argument for its practical implementation stems from its simplicity and translation to the easily recalled public health message, "maintain your waist circumference to less than half your height." Utilizing WHtR, with an exact global boundary value of 0.5, offers a clear advantage for the public health promotion message. The evidence for WHtR as a predictor of CVD and associated risk factors in people with diabetes is emphasized here in the hope that it will encourage the use of this index, particularly in resource-constrained settings, nations with a high burden on the health infrastructure, and countries with a population of mixed ethnicity.
